# Covert pathogenesis: Transient exposures to microbes as triggers of disease

**DOI:** 10.1371/journal.ppat.1007586

**Published:** 2019-03-28

**Authors:** Nicole M. Gilbert, Amanda L. Lewis

**Affiliations:** 1 Department of Obstetrics and Gynecology, Center for Reproductive Health Sciences, Center for Women’s Infectious Disease Research, Washington University School of Medicine, St. Louis, Missouri, United States of America; 2 Department of Molecular Microbiology, Center for Women’s Infectious Disease Research, Washington University School of Medicine, St. Louis, Missouri, United States of America; Nanyang Technological University, SINGAPORE

Our understanding of microbial pathogenesis is founded largely on the assumption that the microbe responsible for causing a disease is the one that is abundantly present at the time and site of disease symptoms. This situation can be compared to the scenario in which a criminal is caught red-handed at the scene of the crime. In this article, we discuss an alternative scenario—“covert pathogenesis”—in which a microbe acts more like a covert operative, sneaking in undetected or unrecognized to trigger disease onset, escaping before the damage is noticed. Here, we will further define “covert pathogenesis,” describe an example of this phenomenon discovered in the urinary tract, highlight other scenarios or diseases that could be impacted by this paradigm, and discuss implications for diagnosis and treatment.

## What is “covert pathogenesis”?

Perhaps the easiest way to define “covert pathogenesis” is to first clarify what it is not. Although covert pathogenesis could involve more than one bacterial species (as in the examples described below), it is different from a polymicrobial infection, in which multiple organisms are present at the same time and in the same location, working in synergy to cause disease. Covert pathogenesis is not simply an alternative term for quiescent latent infection by a recognized primary pathogen and is not to be confused with what is referred to as “covert infection” in the viral field, which encompasses nonproductive latency or persistent low level nonlethal infection [1]. Rather, covert pathogenesis, here, refers to a situation whereby a microbe contributes to disease onset, progression, or severity even though it is not present at the time and place of disease itself. The key feature of covert pathogenesis that distinguishes it from other recognized disease mechanisms is the absence of the covert pathogen at the time and site of disease presentation. In other words, a covert pathogen could never be “caught red-handed” at the scene of the crime.

## Are there known examples of covert pathogens?

The urinary tract is one of the most common sites of infection in humans [2]. *Escherichia coli* is often “caught red-handed” in urine collected at the time patients are experiencing symptoms (increased urination frequency and urgency that is often painful). It is believed that *E*. *coli* gains access to the urinary tract by mechanical transfer from nearby microbial niches. Sexual activity is a leading risk factor for urinary tract infection (UTI), presumably because it aids in the transfer of *E*. *coli* from these nearby niches to the urinary tract [2]. Both the vagina and gastrointestinal tract contain a wealth and diversity of bacterial species. Therefore, when mechanical transfer of bacteria from these niches does occur, the exposure is most certainly polymicrobial in nature. Here, we will discuss our new model that examined the impact of urinary tract exposures to the vaginal bacterium *Gardnerella vaginalis*, which exemplifies the covert pathogenesis paradigm.

**Fig 1:**
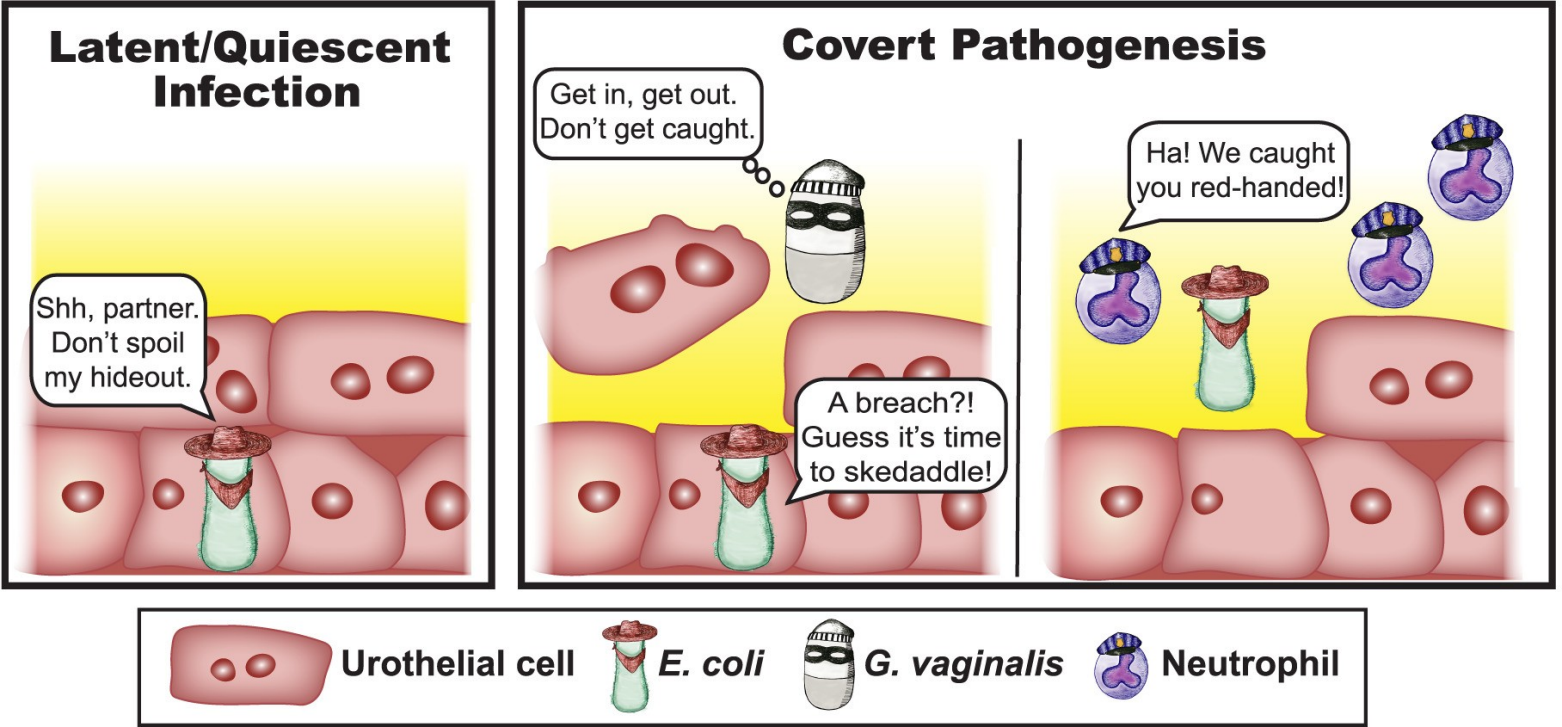
*Gardnerella vaginalis* is a covert pathogen in the bladder. (Left panel) A schematic representation of *Escherichia coli* reservoirs, which become established within bladder epithelial cells during infection in mice. (Right panel) Schematic illustration of what happens when the bladder tissue is exposed to *Gardnerella vaginalis*, namely, that epithelial cells are exfoliated and *E*. *coli* emerges from the epithelium to cause another UTI. Host neutrophils enter the tissue only upon *E*. *coli* emergence, illustrating the concept that *E*. *coli* is “caught red-handed” whereas *G*. *vaginalis* is no longer detectable at the time of a recurrent UTI. UTI, urinary tract infection.

*G*. *vaginalis* is most widely known as a frequent member of the vaginal microbiome, particularly in the context of the dysbiosis known as bacterial vaginosis (BV) [3–6]. *G*. *vaginalis* is a rare cause of symptomatic UTI, but multiple studies have detected (either by culturing or sequencing) *Gardnerella* in urine samples from women [7–10]. Also of note, women with BV have higher rates of UTI than those with a “healthy” lactobacillus-dominated vaginal microbiota [11–14]. Our recent study in mice showed inoculation of the bladder with *G*. *vaginalis* triggered the cells lining the bladder surface to undergo noninflammatory cell death accompanied by exfoliation [15]. Previous studies have shown that, in mice, *E*. *coli* can establish latent infection in intracellular tissue reservoirs in the bladder that can persist for long periods [16–18] (Fig 1, left panel). In humans, sequential UTI episodes are often (up to two-thirds of cases) caused by the same strain of *E*. *coli*, supporting the concept of emergence from a bladder reservoir [19]. Other studies in mice have demonstrated that treatment of the bladder with agents that induce exfoliation of the epithelium can trigger egress of *E*. *coli* to cause recurrent UTI (rUTI) [20–22]. Consistent with these previous studies, our model of bladder exposure to *G*. *vaginalis* in mice harboring *E*. *coli* reservoirs resulted in exfoliation (Fig 1, center panel) and also triggered rUTI marked by *E*. *coli* and neutrophils in urine [15] (Fig 1, right panel). In contrast, bladder exposure to *Lactobacillus crispatus* (widely regarded as a “healthy” vaginal bacterium) caused neither exfoliation nor rUTI. In this mouse model, *G*. *vaginalis* was rapidly cleared (by 12 hours after inoculation) and was no longer present in urine at the time point of *E*. *coli* emergence. Therefore, *G*. *vaginalis* appears to act as a covert pathogen to promote *E*. *coli* rUTI [15].

## Are there other diseases in which covert pathogenesis could be important?

The example of *G*. *vaginalis* reawakening latent *E*. *coli* infection highlights the possibility that other members of the urogenital microbiome could also act as covert pathogens capable of triggering rUTI. Furthermore, the covert pathogenesis paradigm may be involved in reactivation of other latent infections outside of the urinary tract. Current estimates are that nearly one quarter of the world’s population (approximately 1.7 billion people) have latent tuberculosis (TB) infection in their lungs. Although reactivation of TB is more common in people with compromised immunity (HIV+, transplant patients, or those with autoimmune disorders on immunosuppressive drugs) [23], the causes of TB reactivation in otherwise healthy individuals are unclear. We postulate that the covert pathogenesis mechanism could play a role in these cases. The airway is an open system, exposed to a variety of microbes with each breath. Although still somewhat controversial, studies suggest the existence of an airway and/or lung microbiota, which may play roles in immune fitness and various inflammatory and infectious diseases. A few studies have examined the microbiota of sputum comparing patients with active TB to healthy controls [24–27]. Future studies in mouse models could investigate whether covert pathogenesis plays a role in TB reactivation by exposing mice with latent TB to bacteria found in the “airway microbiome,” then monitoring for reactivation of TB infection. Likewise, longitudinal studies in humans could examine whether the presence of certain organisms in sputum precedes the development of active TB.

Covert pathogenesis may represent an emerging paradigm relevant in situations in which the natural triggers of disease have remained obscure, including those that have a long history of being deemed noninfectious because standard culture techniques have failed to consistently find high levels of a single organism. The etiologies of several conditions associated with lower urinary symptoms (bladder pain syndrome, interstitial cystitis, urgency incontinence) fit into this category. The existence of a “urinary microbiome” is becoming more established in the literature, with several studies finding differences in bacterial populations in urine in the context of health versus disease. For example, a recent study detected *G*. *vaginalis* and a handful of other bacterial species more frequently in women with urgency urinary incontinence [9,10]. Even though longitudinal studies have yet to establish whether or not the urinary microbiome represents an established community or reflects the existence of routine transient exposures, the covert pathogenesis model shows that long-standing colonization of the bladder need not be required in order for bacteria to affect disease outcomes.

One can also imagine numerous other situations in which mucosal surfaces (e.g., mouth, throat, gut, vagina) are transiently exposed to microbes found in food, genital secretions, or inhaled particulates, leading to fundamental changes in the body’s interaction with existing microbes or latent pathogens. Furthermore, soluble toxins produced by a microbe at a distant site could also fall under the covert pathogenesis paradigm if the toxin is undetectable at the time and body site of disease presentation. Covert pathogen interactions could also lead to imbalances in the immune response or tissue homeostasis, resulting in inappropriate allergic, inflammatory, autoimmune diseases, or even cancer, even though the inciting microbial exposure is no longer present at the time the disease manifests.

## What are the implications and challenges of covert pathogenesis for diagnosis and/or treatment?

Identification of “covert pathogens” in the context of diseases in humans is challenging because the culprits are absent at the time and site of disease presentation. Therefore, in many instances, a longitudinal study design will be necessary to capture these situations. However, in some cases, a covert pathogen may reside in another bodily reservoir, offering alternative opportunities for diagnosis and intervention. For example, the finding that *G*. *vaginalis* may be a trigger of *E*. *coli* emergence from bladder reservoirs suggests that therapies aimed at reducing *G*. *vaginalis* vaginal colonization may help protect against *E*. *coli* rUTI. An estimated 1% (70 million worldwide) of women suffer more than six rUTIs each year [2]. Preventing rUTI and subsequent pyelonephritis and systemic infection by targeting *G*. *vaginalis* is an exciting concept given the alarming global rise in multidrug resistant *E*. *coli* [28–30]. Such approaches should be feasible because *G*. *vaginalis* is typically sensitive to clindamycin and metronidazole, two drugs that are often used successfully to treat women with BV.

## Conclusions

The human body is constantly being exposed to microbes. Current paradigms in infectious disease pathogenesis typically assume that these exposures are benign unless the microbe takes up residence, multiplies to high levels, and is detectable at the time and in the tissue where symptoms occur. Moving forward, we ought to be vigilant to the possibility that, in some cases, even transient microbial exposures may result in tissue damage or host responses that could result in disease long after a microbe has been cleared.

## References

[1] Williams T, Virto C, Murillo R, Caballero P. Covert Infection of Insects by Baculoviruses. Front Microbiol 2017; 8:1337 10.3389/fmicb.2017.01337 28769903 PMC5511839

[2] Foxman B. Urinary tract infection syndromes: occurrence, recurrence, bacteriology, risk factors, and disease burden. Infect Dis Clin North Am 2014; 28:1–13. 10.1016/j.idc.2013.09.003 24484571

[3] Ravel J, Gajer P, Abdo Z, et al Vaginal microbiome of reproductive-age women. Proc Natl Acad Sci U S A 2011; 108 Suppl 1:4680–7. 20534435 10.1073/pnas.1002611107 PMC3063603

[4] Fredricks DN. Molecular methods to describe the spectrum and dynamics of the vaginal microbiota. Anaerobe 2011; 17:191–5. 10.1016/j.anaerobe.2011.01.001 21376827 PMC3126881

[5] Janulaitiene M, Paliulyte V, Grinceviciene S, et al Prevalence and distribution of Gardnerella vaginalis subgroups in women with and without bacterial vaginosis. BMC Infect Dis 2017; 17:394 10.1186/s12879-017-2501-y 28583109 PMC5460423

[6] Hilbert DW, Schuyler JA, Adelson ME, Mordechai E, Sobel JD, Gygax SE. Gardnerella vaginalis population dynamics in bacterial vaginosis. Eur J Clin Microbiol Infect Dis 2017; 36:1269–78. 10.1007/s10096-017-2933-8 28197729

[7] Lam MH, Birch DF, Fairley KF. Prevalence of Gardnerella vaginalis in the urinary tract. J Clin Microbiol 1988; 26:1130–3. 3260242 10.1128/jcm.26.6.1130-1133.1988 PMC266547

[8] Wolfe AJ, Toh E, Shibata N, et al Evidence of uncultivated bacteria in the adult female bladder. J Clin Microbiol 2012; 50:1376–83. 10.1128/JCM.05852-11 22278835 PMC3318548

[9] Pearce MM, Hilt EE, Rosenfeld AB, et al The female urinary microbiome: a comparison of women with and without urgency urinary incontinence. MBio 2014; 5:e01283–14. 10.1128/mBio.01283-14 25006228 PMC4161260

[10] Pearce MM, Zilliox MJ, Rosenfeld AB, et al The female urinary microbiome in urgency urinary incontinence. Am J Obstet Gynecol 2015; 213:347 e1–11. 26210757 10.1016/j.ajog.2015.07.009 PMC4556587

[11] Amatya R, Bhattarai S, Mandal PK, Tuladhar H, Karki BM. Urinary tract infection in vaginitis: a condition often overlooked. Nepal Med Coll J 2013; 15:65–7. 24592798

[12] Harmanli OH, Cheng GY, Nyirjesy P, Chatwani A, Gaughan JP. Urinary tract infections in women with bacterial vaginosis. Obstet Gynecol 2000; 95:710–2. 10775734 10.1016/s0029-7844(99)00632-8

[13] Hillebrand L, Harmanli OH, Whiteman V, Khandelwal M. Urinary tract infections in pregnant women with bacterial vaginosis. Am J Obstet Gynecol 2002; 186:916–7. 12015512 10.1067/mob.2002.123987

[14] Sharami SH, Afrakhteh M, Shakiba M. Urinary tract infections in pregnant women with bacterial vaginosis. J Obstet Gynaecol 2007; 27:252–4. 10.1080/01443610701194846 17464804

[15] Gilbert NM, O'Brien VP, Lewis AL. Transient microbiota exposures activate dormant Escherichia coli infection in the bladder and drive severe outcomes of recurrent disease. PLoS Pathog 2017; 13:e1006238 10.1371/journal.ppat.1006238 28358889 PMC5373645

[16] Kerrn MB, Struve C, Blom J, Frimodt-Moller N, Krogfelt KA. Intracellular persistence of Escherichia coli in urinary bladders from mecillinam-treated mice. J Antimicrob Chemother 2005; 55:383–6. 10.1093/jac/dki002 15681580

[17] Mulvey MA, Schilling JD, Hultgren SJ. Establishment of a persistent Escherichia coli reservoir during the acute phase of a bladder infection. Infect Immun 2001; 69:4572–9. 10.1128/IAI.69.7.4572-4579.2001 11402001 PMC98534

[18] Mulvey MA, Schilling JD, Martinez JJ, Hultgren SJ. Bad bugs and beleaguered bladders: interplay between uropathogenic Escherichia coli and innate host defenses. Proc Natl Acad Sci U S A 2000; 97:8829–35. 10922042 10.1073/pnas.97.16.8829 PMC34019

[19] Silverman JA, Schreiber HLt, Hooton TM, Hultgren SJ. From physiology to pharmacy: developments in the pathogenesis and treatment of recurrent urinary tract infections. Curr Urol Rep 2013; 14:448–56. 10.1007/s11934-013-0354-5 23832844 PMC3797163

[20] Eto DS, Sundsbak JL, Mulvey MA. Actin-gated intracellular growth and resurgence of uropathogenic Escherichia coli. Cell Microbiol 2006; 8:704–17. 10.1111/j.1462-5822.2006.00691.x 16548895

[21] Mysorekar IU, Hultgren SJ. Mechanisms of uropathogenic Escherichia coli persistence and eradication from the urinary tract. Proc Natl Acad Sci U S A 2006; 103:14170–5. 10.1073/pnas.0602136103 16968784 PMC1564066

[22] Blango MG, Ott EM, Erman A, Veranic P, Mulvey MA. Forced resurgence and targeting of intracellular uropathogenic Escherichia coli reservoirs. PLoS ONE 2014; 9:e93327 10.1371/journal.pone.0093327 24667805 PMC3965547

[23] Kiazyk S, Ball TB. Latent tuberculosis infection: An overview. Can Commun Dis Rep 2017; 43:62–6. 29770066 10.14745/ccdr.v43i34a01 PMC5764738

[24] Cui Z, Zhou Y, Li H, et al Complex sputum microbial composition in patients with pulmonary tuberculosis. BMC Microbiol 2012; 12:276 10.1186/1471-2180-12-276 23176186 PMC3541192

[25] Cheung MK, Lam WY, Fung WY, et al Sputum microbiota in tuberculosis as revealed by 16S rRNA pyrosequencing. PLoS ONE 2013; 8:e54574 10.1371/journal.pone.0054574 23365674 PMC3554703

[26] Wu J, Liu W, He L, et al Sputum microbiota associated with new, recurrent and treatment failure tuberculosis. PLoS ONE 2013; 8:e83445 10.1371/journal.pone.0083445 24349510 PMC3862690

[27] Krishna P, Jain A, Bisen PS. Microbiome diversity in the sputum of patients with pulmonary tuberculosis. Eur J Clin Microbiol Infect Dis 2016; 35:1205–10. 10.1007/s10096-016-2654-4 27142586

[28] Malekzadegan Y, Khashei R, Sedigh Ebrahim-Saraie H, Jahanabadi Z. Distribution of virulence genes and their association with antimicrobial resistance among uropathogenic Escherichia coli isolates from Iranian patients. BMC Infect Dis 2018; 18:572 10.1186/s12879-018-3467-0 30442101 PMC6238375

[29] Paniagua-Contreras GL, Monroy-Perez E, Bautista A, et al Multiple antibiotic resistances and virulence markers of uropathogenic Escherichia coli from Mexico. Pathog Glob Health 2018:1–6. 10.1080/20477724.2018.1547542 PMC6327565 30433859

[30] Walker E, Lyman A, Gupta K, Mahoney MV, Snyder GM, Hirsch EB. Clinical Management of an Increasing Threat: Outpatient Urinary Tract Infections Due to Multidrug-Resistant Uropathogens. Clin Infect Dis 2016; 63:960–5. 10.1093/cid/ciw396 27313263

